# Neurophysiological correlates of altered response inhibition in internet gaming disorder and obsessive-compulsive disorder: Perspectives from impulsivity and compulsivity

**DOI:** 10.1038/srep41742

**Published:** 2017-01-30

**Authors:** Minah Kim, Tak Hyung Lee, Jung-Seok Choi, Yoo Bin Kwak, Wu Jeong Hwang, Taekwan Kim, Ji Yoon Lee, Jae-A Lim, Minkyung Park, Yeon Jin Kim, Sung Nyun Kim, Dai Jin Kim, Jun Soo Kwon

**Affiliations:** 1 Department of Psychiatry, Seoul National University College of Medicine, Seoul, Republic of Korea; 2 Department of Brain and Cognitive Science, Seoul National University College of Natural Science, Seoul, Republic of Korea; 3 Department of Psychiatry, SMG-SNU Boramae Medical Center, Seoul, Republic of Korea; 4 Interdisciplinary program in Neuroscience, Seoul National University College of Natural Science, Seoul, Republic of Korea; 5 Department of Psychiatry, Seoul St. Mary’s Hospital, The Catholic University of Korea College of Medicine, Seoul, Republic of Korea

## Abstract

Although internet gaming disorder (IGD) and obsessive-compulsive disorder (OCD) represent opposite ends of the impulsivity and compulsivity dimensions, the two disorders share common neurocognitive deficits in response inhibition. However, the similarities and differences in neurophysiological features of altered response inhibition between IGD and OCD have not been investigated sufficiently. In total, 27 patients with IGD, 24 patients with OCD, and 26 healthy control (HC) subjects participated in a Go/NoGo task with electroencephalographic recordings. N2-P3 complexes elicited during Go and NoGo condition were analyzed separately and compared among conditions and groups. NoGo-N2 latency at the central electrode site was delayed in IGD group versus the HC group and correlated positively with the severity of internet game addiction and impulsivity. NoGo-N2 amplitude at the frontal electrode site was smaller in OCD patients than in IGD patients. These findings suggest that prolonged NoGo-N2 latency may serve as a marker of trait impulsivity in IGD and reduced NoGo-N2 amplitude may be a differential neurophysiological feature between OCD from IGD with regard to compulsivity. We report the first differential neurophysiological correlate of the altered response inhibition in IGD and OCD, which may be a candidate biomarker for impulsivity and compulsivity.

Historically, classification models of psychiatric illnesses have placed impulsive disorders and compulsive disorders on opposite ends of a single dimension^1^. Most representative impulsive disorders are addictive disorders, such as pathological gambling (PG) or substance dependence, which show risk-taking behavior for immediate gratification as a core characteristic^2^,^3^. On the other hand, obsessive-compulsive disorder (OCD) has been considered the most classic form of compulsive disorder because compulsions in OCD are believed to be rather stereotypic, often ego-dystonic, and focused on harm avoidance^4^,^5^. Despite this, recent reports have focused on the similarities between impulsive and compulsive disorders, such as deficits in response inhibition, brain circuitry, and comorbidities, suggesting that impulsivity and compulsivity are orthogonal factors that each contribute, to varying degrees, to various psychiatric conditions^6^,^7^. From this point of view, the American Psychiatric Association provided a new obsessive-compulsive and related disorders (OCRD) category in the Diagnostic and Statistical Manual of Mental Disorders, 5^th^ edition (DSM-5), in which similarities and differences in impulsive and compulsive disorders could be compared and further investigated from multiple perspectives^6^.

Internet gaming disorder (IGD) is classified as a behavioral addiction, characterized by an inability to control internet game usage despite functional impairment, similar to gambling in PG^8^,^9^. With the popularization of the internet and the rapid growth in its game industry, individuals with IGD have increased in number and shown tendencies toward various psychiatric comorbidities^10^,^11^,^12^,^13^. Reflecting the emerging clinical interest in IGD, section 3 of DSM-5 (Emerging Measures and Models) included this condition, along with a list of proposed diagnostic criteria to encourage future research^14^. Impulsivity and a failure of inhibitory control in IGD have been suggested using various modalities, such as behavioral, electrophysiological, and functional neuroimaging paradigms^15^,^16^,^17^. Impaired response inhibition has also been reported in OCD, in accordance with obsessive-compulsive symptom severity and inefficient top-down regulation^18^,^19^. Deficits in response inhibition may be caused by different neural responses, in terms of impulsivity or compulsivity, to the shared urge to perform a specific act^20^,^21^. Thus, investigating the neurobiological correlate(s) of altered response inhibition in IGD and OCD may be helpful in understanding the role of impulsivity and compulsivity in psychiatric disorders.

The N2 and P3 event-related potential (ERP) components in Go/NoGo tasks have been conceptualized as neurophysiological correlates of response inhibition^22^. In healthy individuals, withholding a response to a NoGo stimulus produces a larger N2-P3 complex than does responding to a Go stimulus, indicating that NoGo-N2 and -P3 reflects the process of inhibitory control^23^. Previous research has suggested that NoGo-N2 reflects an early stage of inhibitory control or conflict monitoring^24^,^25^,^26^. The other ERP component, NoGo-P3, may represent a later stage of the inhibitory process in both the cognitive and motor domains^27^,^28^. Regarding both the NoGo-N2 and -P3 components in healthy subjects, amplitude has been suggested as a marker of either successful inhibition or the subjective effort required to inhibit a response, and latency has been considered to reflect the latter^22^,^29^.

Although there have been several studies on response inhibition in IGD using a Go/NoGo paradigm, results have not been consistent across studies. Two studies suggested that NoGo-N2 amplitudes of excessive internet users were reduced, perhaps due to a mediating effect of the associated impulsivity. However, since no correlations were observed between the NoGo-N2 amplitude and any measure of impulsivity in these studies, markers of trait impulsivity in IGD subjects could not be identified^17^,^30^. In contrast, two other studies reported increased NoGo-N2 amplitudes in excessive gamers or smartphone users and interpreted the results as compensatory hyperactivity for response inhibition failure^31^,^32^. These inconsistencies may be due to variation in task difficulty among studies, which is known to have an effect on the direction of NoGo-N2 amplitude alteration (i.e., enhanced or decreased)^33^. Regarding NoGo-P3, only the study of Dong *et al*. reported a significant group difference in NoGo-P3 amplitude and latency^17^. Previous ERP studies in OCD patients using Go/NoGo tasks or Stop Signal Tasks (SST) assessed the relationship between response inhibition and compulsivity. Kim *et al*. showed that NoGo-N2 amplitudes at fronto-central sites were reduced and were negatively associated with obsessive-compulsive symptom severity^18^. In another study, Hermann *et al*. showed that OCD patients had reduced frontal activity during the NoGo condition, and that anteriorization was negatively correlated with Yale-Brown obsessive compulsive scale (Y-BOCS) scores^34^. Johannes *et al*., on the other hand, found that Stop-N2 amplitude was increased in OCD patients during SST performance^35^. In addition, Lei *et al*. reported that increased Stop-N2 amplitude was a general feature in the OCD patients regardless of symptom dimension and not correlated with OC symptom severity^36^.

Despite the growing interest in identifying the pathophysiological and neurobiological mechanisms of IGD and OCD in terms of the impulsivity and compulsivity spectra, no study to date has directly compared the neurophysiological correlate(s) of response inhibition in IGD versus OCD. Furthermore, studies including IGD subjects have reported inconsistent results, which may be due to differences in task complexity among studies; furthermore, no significant neurophysiological correlate of impulsivity has been identified^17^,^30^,^31^,^32^. In the current study, we investigated the similarities and differences in response inhibition of IGD versus OCD during Go/NoGo task performance. We measured both behavioral and neurophysiological aspects of response inhibition and used tasks of equal difficulty in each group to control for any possible effect of task complexity on ERP responses. We first hypothesized that individuals with IGD and patients with OCD would show similar deficits in response inhibition, as indexed by behavioral performance. Second, we expected any failure in inhibitory control, in IGD or OCD, to be related to different neurophysiological features between the disorders with respect to impulsivity and compulsivity.

## Results

### Demographics, clinical characteristics, and Go-NoGo behavioral data

We found no significant group difference in sex, handedness, IQ, or education (Table 1). Scores on the IAT (F_2,72_ = 24.702, p < 0.001), BIS-11 (F_2,72_ = 4.209, p = 0.019), BDI (F_2,72_ = 11.557, p < 0.001), and BAI (F_2,72_ = 10.507, p = 0.001) were significantly different among the groups. Participants with IGD showed the highest scores on the IAT, patients with OCD were intermediate, and healthy control (HC) subjects showed the lowest scores (IGD vs. HC, p < 0.001, IGD vs. OCD, p < 0.001, OCD vs. HC, p = 0.028). Impulsiveness, as indexed by the BIS-11 score, was higher in the IGD group than in the HC group (p = 0.019). However, differences in BIS-11 scores were not significant between the HC and OCD groups (p = 0.106), or between the IGD and OCD groups (p = 0.826). Both IGD and OCD subjects showed more severe depressive and anxiety symptoms, as shown by their BDI (IGD vs. HC, p = 0.006, OCD vs. HC, p < 0.001) and BAI (IGD vs. HC, p = 0.020, OCD vs. HC, p < 0.001) scores, than the HCs.

The RTs in the Go trial did not differ significantly among the groups. Although the IGD group responded more rapidly, and the OCD group more slowly, than the other two groups, no statistically significant group difference was observed. However, the ER in the NoGo trial (errors of commission) did differ significantly among the groups (F = 4.242, p = 0.018); the HCs showed a lower ER than the IGD (p = 0.031) and OCD (p = 0.044) participants.

### ERP amplitudes and latencies

**Figure 1:**
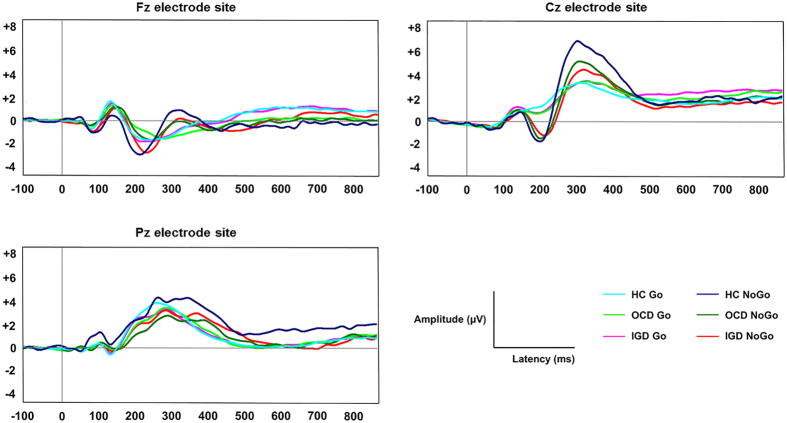
Grand-averaged event-related potential waveforms of Go/NoGo conditions across the three groups at the Fz, Cz, and Pz electrode sites.

Figure 1 shows the grand-averaged ERP waveforms at the Fz, Cz, and Pz electrode sites. There were significant main effects of inhibitory condition (Go/NoGo) on N2 amplitude (F_1,74_ = 59.594, p < 0.001) and latency (F_1,74_ = 6.902, p = 0.010), as well as in P3 amplitude (F_1,74_ = 48.469, p < 0.001) and latency (F_1,74_ = 4.229, p = 0.043). There was no significant group by inhibitory condition interaction effect on N2 amplitude (F_1,74_ = 2.628, p = 0.079) or latency (F_1,74_ = 2.071, p = 0.133), or on P3 amplitude (F_1,74_ = 0.030, p = 0.971) or latency (F_1,74_ = 0.681, p = 0.509). Indeed, all three groups showed larger N2 and P3 amplitudes, and longer N2 and P3 latencies in NoGo than in Go trials. Repeated-measures ANOVA with electrode site (six fronto-central electrodes for N2 and six centro-parietal electrodes for P3) as the within-subject factor and group (IGD/OCD/HC) as a between-subjects factor revealed a significant main effect of group on NoGo-N2 latency (F_2,74_ = 3.880, uncorrected p = 0.025). After applying Bonferroni correction for multiple repeated-measures ANOVAs, main effect of group on NoGo-latency showed trend level significance that indicated intermediate effect (corrected p = 0.100). There was a significant effect of electrode site on NoGo-N2 latency (F_5,70_ = 17.652, p < 0.001) and NoGo-N2 amplitude (F_5,70_ = 16.364, p < 0.001). A *post hoc* Bonferroni test showed that NoGo-N2 latency was prolonged in IGD subjects (p = 0.025) compared to that in HCs, whereas no difference was found between the IGD and OCD groups (p = 1.000) or between the OCD and HC groups (p = 0.191). No significant group effect was seen in any of the other variables (Go-N2 amplitude, F_2,74_ = 0.152, p = 0.859, Go-N2 latency, F_2,74_ = 1.860, p = 0.163, Go-P3 amplitude, F_2,74_ = 0.134, p = 0.875, Go-P3 latency, F_2,74_ = 3.880, p = 0.025, NoGo-N2 amplitude, F_2,74_ = 2.111, p = 0.128, NoGo-P3 amplitude, F_2,74_ = 0.057, p = 0.945, NoGo-P3 latency, F_2,74_ = 1.927, p = 0.153). Table 2 summarizes the means (standard deviations) of Go- and NoGo-N2 amplitudes and latencies at each electrode site, and the results of the group comparison. Patients with OCD showed reduced NoGo-N2 amplitudes at F2 compared to individuals with IGD, after Bonferroni correction (uncorrected p = 0.006, corrected p = 0.036). There was no group difference in NoGo-N2 amplitude at F2 between the IGD and HC groups (p = 0.469) or between the OCD and HC groups (p = 0.123). Table 3 presents the means (standard deviations) of Go- and NoGo-P3 amplitudes and latencies at each electrode site, and the results of the group comparison. Compared to HCs, OCD patients showed longer Go-P3 latencies at the C1 electrode site (uncorrected p = 0.024, corrected p = 0.144), while subjects with IGD showed prolonged Go-P3 latencies at P1 (uncorrected p = 0.028, corrected p = 0.168) and NoGo-P3 latencies at Cz (uncorrected p = 0.029, corrected p = 0.174). However, these statistical differences did not survive after Bonferroni correction.

### Correlation analysis

**Figure 2:**
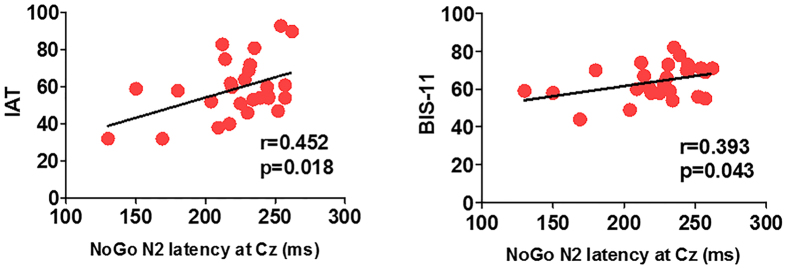
Correlation of the NoGo-N2 latency at the Cz electrode site with scores on the Korean version of Young’s Internet Addiction Test (IAT) and the Barratt Impulsiveness Scale version 11 (BIS-11) in individuals with internet gaming disorder.

Pearson’s correlation analysis was performed for NoGo-N2 latency at Cz, NoGo-N2 latency at C2, IAT scores, BIS-11 scores in the IGD group; and for NoGo-N2 amplitude at F2, Y-BOCS total scores, obsession scores, and compulsion scores in the OCD group. Significant relationships between NoGo-N2 latency at Cz and IAT scores (r = 0.452, p = 0.018) and BIS-11 scores (r = 0.393, p = 0.043) were found in the IGD group (Fig. 2). NoGo-N2 latency at C2 correlated with neither IAT scores (r = 0.057, p = 0.777) nor BIS-11 scores (r = 0.170, p = 0.398) in the IGD group. In the OCD group, no significant relationship was found between NoGo-N2 amplitude at F2 and Y-BOCS total scores (r = −0.192, p = 0.370), obsession scores (r = −0.252, p = 0.235), or compulsion scores (r = −0.091, p = 0.674).

## Discussion

To our knowledge, this is the first reported investigation of different neurophysiological correlates of response inhibition in IGD and OCD. As hypothesized, IGD and OCD participants showed increased ERs in the NoGo condition (errors of commission), indicating that both the IGD and OCD groups showed difficulties in response inhibition at the behavioral level. Regarding the neurophysiological findings, all three groups showed larger N2-P3 amplitudes and longer N2-P3 latencies in the NoGo than in the Go condition. Delayed NoGo-N2 latency at a central site was found in the IGD group versus HCs with intermediate effect, and correlated positively with internet game addiction severity and impulsivity scores. The NoGo-N2 amplitude at the frontal site was reduced in OCD patients versus IGD individuals; however, the correlation between NoGo-N2 amplitude at the frontal site and obsessive-compulsive symptom severity was not significant.

Consistent with previous studies, IGD subjects showed the highest levels of impulsivity, as indexed by BIS-11 scores, among the groups^37^,^38^. Latency of the N2-P3 complex in the NoGo condition is regarded as the cognitive demand required to monitor conflict and inhibit responses successfully^29^. Benikos *et al*. reported that NoGo-N2 amplitude was enhanced with increasing task difficulty and subjective effort to inhibit responses^33^. It has also been shown that psychiatric conditions with high impulsivity, such as attention-deficit and hyperactivity disorder, borderline personality disorder, and psychopathy, exhibit altered NoGo N2-P3 complexes^39^,^40^,^41^. In the current study, the NoGo-N2 amplitude was larger in IGD individuals than in OCD patients, suggesting that despite the shared inhibitory control deficits, there are differences in the neurophysiological correlates of impulsivity and compulsivity between these two populations. In addition, NoGo-N2 latency in IGD individuals was delayed compared to that in HC subjects, indicating that IGD subjects had difficulty with response inhibition in the early stages, thus requiring more cognitive resources. Furthermore, the severity of IGD and impulsivity correlated positively with NoGo-N2 latency at the central site, suggesting that a failure of inhibitory control in IGD subjects may be related to increased cognitive demand for response inhibition, due to their higher impulsivity.

Previous studies reported that repeated behaviors in OCD are more compulsive than impulsive, because OCD patients show a relatively preserved capacity to delay a reward, unlike addiction patients^42^,^43^. Similarly, we found less prominent impulsivity in OCD patients versus IGD subjects. Moreover, OCD patients showed smaller NoGo-N2 amplitudes at the frontal site than IGD individuals, indicating that NoGo-N2 amplitude in OCD may reflect dysfunction in frontal region(s) that inhibit(s) compulsive behaviors^18^. According to source analysis results of previous studies, the NoGo-N2 component originates from the medial orbitofrontal and cingulate cortices^22^,^44^. These regions have been reported to be the neural correlates of response inhibition in a study using functional magnetic resonance imaging^21^. In OCD patients, the regions in the ventral cognitive circuit of the cortico-striato-thalamo-cortical loop known to mediate motor and response inhibition have been suggested to be the neural correlates of obsessive-compulsive symptoms^45^,^46^. Taking these findings together, reduced NoGo-N2 amplitude at the frontal site in our group of OCD patients may reflect dysfunction in the neurophysiological correlates of inhibitory control, mediated by frontal cortical regions.

Contrary to results reported by previous studies, we found no significant difference in NoGo-N2 amplitude between OCD patients and HC subjects^18^,^34^,^35^,^36^,^47^. Previous literature on NoGo- or Stop-N2 in OCD patients reported opposite direction of N2 amplitude (increased or decreased) with regard to study design. Studies that reported smaller NoGo-N2 in OCD patients than in HCs used Go/NoGo task without oddball paradigm and interpreted their findings as the reflection of impaired response inhibition^18^,^34^. Studies that reported larger Stop-N2 in OCD patients, on the other hand, used Go/NoGo task with complex oddball paradigm or SST and suggested that increased cognitive demand in performing response inhibition enlarged NoGo- or Stop-N2^35^,^36^,^47^. It has been suggested that NoGo- or Stop-N2 showed a similar topography and estimated source location as error-related negativity, and the NoGo- or Stop-N2 has been found to be largest under high conflict conditions^47^. Thus, NoGo- or Stop-N2 component may be involved in situations wherein responsive conflict is high. The Go/NoGo task used in the current study included simple oddball paradigm that was not included in the previous studies reporting reduced NoGo-N2 in OCD patients^18^,^34^ and, furthermore, accompanied relatively low conflict condition compared to SST used in the Lei *et al*. study, which reported increased Stop-N2 amplitude^36^. Therefore, the intermediate conflict condition produced by the Go/NoGo task in this study may have elicited intermediate NoGo-N2 amplitude in OCD patients that may, in turn, have blurred the contrast between OCD and HC groups.

In this study, both IGD and OCD participants showed behavioral deficits in response inhibition, as assessed by an increased ER during the Go/NoGo task. However, the neural response to withholding behavioral responses to the NoGo stimuli differed between the groups, suggesting different neurophysiological correlates of altered response inhibition. Although failure of inhibitory control can result from both impulsivity and compulsivity, the process of impulsivity is related to the tendency to act on impulse, whereas compulsivity is related to a problem in terminating actions^7^,^48^. Specifically, we found that NoGo-N2 amplitude at the frontal site was increased in the IGD group, whereas the OCD group showed a relative decrease in NoGo-N2 amplitude during performance of the same Go/NoGo task. Previous ERP studies using Go/NoGo tasks have reported inconsistent results regarding the direction (enhanced or reduced) of NoGo-N2 amplitude, possibly due to the combined effect of subjective effort and differences in the degree of task difficulty among different Go/NoGo paradigms^29^,^33^,^49^. Thus, our finding of group difference in NoGo-N2 amplitude between IGD and OCD may reflect different neural responses, mediated by group differences in the subjective effort required for inhibitory control during performance of the same Go/NoGo task.

This study had several limitations. First, although we recruited OCD patients with compulsive symptoms, NoGo-N2 amplitudes at the frontal site did not correlate significantly with scores on the Y-BOCS. Thus, without using the analogical inference, it is unclear whether the reduced NoGo-N2 amplitude at the frontal site in OCD patients directly represents a neurophysiological correlate of compulsivity. Second, many of the IGD patients in our study were not seeking treatment and their addiction was less severe (mean IAT score <60) compared to that of participants in previous studies. Additionally, the OCD patients in this study were somewhat heterogeneous, so their medication status and comorbidities could not be controlled-for in the analysis of ERPs. Those heterogeneities may have reduced the ERP contrast among the three groups; however, despite the heterogeneity, the results do support the hypothesis, so long as a cautious interpretation is maintained. Third, group difference of NoGo-N2 latency showed intermediate effect after applying correction for multiple comparisons, and correction for multiple tests was not performed for the correlation analyses. Therefore, caution should be warranted in interpreting the results of the current study in relations to clinical efficacy.

We sought to investigate the different neurophysiological correlates of dysfunctional response inhibition in IGD and OCD, using a Go/NoGo paradigm, in terms of both impulsivity and compulsivity. Behavioral data indicated that both IGD and OCD patients had difficulties in response inhibition. ERP results demonstrated that individuals with IGD had more demand for cognitive control in the early stages of response inhibition, according to addiction severity and the degree of impulsivity. In patients with OCD, it could be that the deficits in response inhibition reflect dysfunction in the frontal cortex, which was related to the inhibitory control of compulsive behavior. Taken together, the delayed NoGo-N2 latency may be a biomarker of trait impulsivity in IGD patients, and the reduced NoGo-N2 amplitude may serve as a differential neurophysiological feature in OCD versus IGD in association with compulsivity. Future studies with more homogeneous samples, and a Go/NoGo paradigm better suited to a direct comparison of IGD versus OCD, are needed to extend and confirm the findings of the current study.

## Methods

### Participants and Clinical assessments

In total, 27 subjects with IGD, 24 patients with OCD, and 26 HC subjects participated in this study. The IGD subjects were recruited from the addiction outpatient clinic of SMG-SNU Boramae Medical Center, as well as via an advertisement. The HC subjects were recruited via an online advertisement. OCD patients were recruited from the OCD outpatient clinic at Seoul National University Hospital (SNUH). All subjects with IGD participated in internet gaming for >4 h/day and were medication-naïve. An experienced psychiatrist performed interviews to confirm the diagnoses of IGD and OCD using the DSM-5 criteria. Considering the study purpose, of investigating impulsivity and compulsivity, only patients with OCD who had compulsive symptoms were included. Seven OCD patients were medication-naïve, ten were medication-free for >1 month before entering the study, and seven were medicated at the time of testing. The seven medicated OCD patients were taking selective serotonin reuptake inhibitors, and one patient was prescribed a small dose of olanzapine (2.5 mg) as an adjuvant. The severity of OCD was assessed using the Y-BOCS^50^. HC subjects played internet games for <2 h/day and reported no past or current psychiatric illness. In all participants, Young’s Internet Addiction Test (IAT)^51^ and the Barratt Impulsiveness Scale (BIS-11)^52^ were used to measure the severity of internet gaming addiction and the degree of impulsivity. Depressive and anxiety symptoms were assessed using the Beck Depression Inventory (BDI)^53^ and the Beck Anxiety Inventory (BAI)^54^. The intelligence quotient (IQ) was measured using the abbreviated version of the Korean-Wechsler Adult Intelligence Scale. Exclusion criteria included a lifetime diagnosis of substance abuse or dependence, neurological disease, significant head injury accompanied by loss of consciousness, any medical illness with documented cognitive sequelae, sensory impairments, and intellectual disability (IQ < 70).

All of the participants fully understood the study procedure and provided written informed consent. The study was conducted in accordance with the Declaration of Helsinki. The institutional review boards of SMG-SNU Boramae Medical Center and SNUH approved the study.

### Go/Nogo Task and EEG recordings

Participants were seated comfortably in a dimly lit, electrically shielded room, ~60 cm away from a monitor on which a pseudo-random series of 300-ms visual stimuli, “S” and “O”, were presented. Subjects were instructed to respond with a button press to the frequent “S” stimulus (Go trial, 71.4%, 428/600) and not to respond to the infrequent “O” stimulus (NoGo trial, 28.6%, 172/600). The inter-trial interval was 1,500 ms. Continuous electroencephalogram (EEG) recordings were made using a Neuroscan 128-channel Synamps system with a 128-channel Quick-Cap, based on the modified 10–20 international system (Compumedics, Charlotte, NC, USA). The electrodes at the mastoid sites served as reference electrodes and the ground electrode was placed between the FPz and Fz electrode sites. The EEG was digitized at a 1,000-Hz sampling rate with an online filter of 0.05 to 100 Hz. Eye movement artifacts were monitored by recording the vertical and horizontal electro-oculogram (EOG) using electrodes below, and on the outer canthus of, the left eye. The resistance at all electrode sites was below 5 kΩ.

### ERP analysis

Offline processing of ERP data was performed using the Curry software (ver. 7; Compumedics, Charlotte, NC, USA). Eye movement artifacts were reduced using the ocular artifact-reduction algorithm, which regresses eye-blink activity based on the vertical EOG signal^55^. The threshold used for the vertical EOG signal was 200 μV. Time intervals of 200 ms before and 500 ms after threshold detection were used for the regression. Continuous EEG recordings were re-referenced to a common average reference, bandpass filtered between 0.1 Hz and 30 Hz, epoched to 100 ms pre-stimulus and 900 ms post-stimulus, and baseline-corrected using the averaged pre-stimulus interval voltage. Epochs containing EEG amplitudes that exceeded ± 75 μV were rejected automatically. Importantly, analysis of variance (ANOVA) revealed that the number of epochs remaining after the artifact rejection procedure did not differ among the three groups (Go, F_2,76_ = 0.508, p = 0.604; NoGo, F_2,76_ = 1.355, p = 0.264). The mean (standard deviation) of the number of remaining epochs in the Go condition was 343.8 (67.9) in HCs, 327.9 (82.0) in the IGD group, and 347.3 (71.4) in the OCD group. The corresponding values in the NoGo condition were 132.9 (28.6) in the HCs, 118.9 (34.8) in the IGD group, and 121.0 (35.4) in the OCD group. The epochs were then averaged separately for each condition (Go vs. NoGo). A peak detection method was used to determine the Go- and NoGo-N2 peak amplitudes and latencies, which were defined as the amplitudes showing the most negative deflection between 130 ms and 280 ms post-stimulus onset at the frontal (F1, Fz, F2) and central (C1, Cz, C2) electrode sites. The Go- and NoGo-P3 peak amplitudes and latencies were defined as those showing the most positive deflection between 250 ms and 450 ms post-stimulus onset at the central (C1, Cz, C2) and parietal (P1, Pz, P2) electrode sites. Channels and peak detection time windows were included in the analysis according to previous reports on the locations of the most prominent N2 and P3 amplitudes (in terms of channel location and time range)^29^,^56^.

### Statistical analysis

The demographic and clinical characteristics of the subjects were compared among groups using one-way ANOVA, independent sample t-tests, or Welch’s test if the variances were not equal. A χ^2^ analysis or Fisher’s exact test was used for categorical data analysis. ANOVAs were performed to test for a group difference in the reaction time (RT) in Go trials, and the error rate (ER) in NoGo trials. Inhibitory effects on ERP amplitudes and latencies were analyzed using repeated-measures ANOVA with electrode sites (F1, Fz, F2, C1, Cz, C2 for N2/C1, Cz, C2, P1, Pz, P2 for P3) and stimuli (Go/NoGo) as within-subject factors and group (IGD/OCD/HC) as a between-subjects factor. Group comparisons of ERP amplitude and latency were performed using repeated-measures ANOVA with electrode site (six fronto-central electrodes for N2, six centro-parietal electrodes for P3) as the within-subject factor and group (IGD/OCD/HC) as a between-subjects factor. A *post hoc* Bonferroni test was used to test for pairwise differences. Pearson’s correlation was used to assess the relationship among ERP amplitude and latencies that showed a group difference, as well as IAT scores, BIS-11 scores within the IGD group, and Y-BOCS scores within the OCD group. For the correlation analyses, correction for multiple tests was not applied, because the analyses were considered exploratory in nature. SPSS software (ver. 22.0; IBM Corp., Armonk, NY, USA) was used for statistical analyses. P values < 0.05 were considered to indicate statistical significance.

## Additional Information

**How to cite this article:** Kim, M. *et al*. Neurophysiological correlates of altered response inhibition in internet gaming disorder and obsessive-compulsive disorder: Perspectives from impulsivity and compulsivity. *Sci. Rep.* **7**, 41742; doi: 10.1038/srep41742 (2017).

**Publisher's note:** Springer Nature remains neutral with regard to jurisdictional claims in published maps and institutional affiliations.

## Figures and Tables

**Table 1:** Demographics, clinical characteristics, and Go/NoGo behavior of participants.

	HC	IGD	OCD	Statistical analysis^a^	
	(N = 26)	(N = 27)	(N = 24)	χ^2^, *F* or *T*	*P*
Demographics					
Sex (Male/Female)	18/8	24/3	19/5	3.109	0.211
Handedness (Right/Left)	25/1	25/2	23/1	0.416	0.812
Age (years)	24.7 (4.7)	26.5 (6.1)	25.0 (5.7)	0.801	0.453
IQ	117.5 (7.7)	110.4 (15.0)	110.0 (15.6)	2.639	0.078
Education (years)	13.7 (1.3)	14.6 (1.8)	13.8 (2.0)	2.055	0.135
Clinical characteristics					
Y-IAT-K^b^	31.3 (8.8)	59.1 (15.9)	42.3 (17.8)	24.702	<0.001**
BIS-11^b^	57.4 (7.6)	64.0 (9.0)	62.5 (9.2)	4.209	0.019*
BDI^b^	4.4 (4.2)	11.5 (9.0)	15.4 (10.2)	11.557	<0.001**
BAI^b^	2.7 (3.5)	10.2 (11.2)	15.4 (12.5)	10.507	<0.001**
Y-BOCS Total	NA	NA	22.4 (6.9)	NA	NA
Obsession	NA	NA	11.3 (4.0)	NA	NA
Compulsion	NA	NA	11.1 (3.4)	NA	NA
Go/NoGo behavior					
Reaction time (ms)^c^	263.3 (37.0)	255.2 (34.2)	287.7 (131.0)	1.153	0.321
Error rate (%)^d^	8.3 (5.8)	14.8 (11.1)	14.7 (9.8)	4.242	0.018*

^a^Analysis of variance or Independent t test or Welch’s t test if the variances were not equal, χ^2^ analysis or Fisher’s exact test for categorical data.

^b^With 2 missing values in OCD group.

^c^Behavioral reaction time in the Go trial.

^d^Error rate in the NoGo trial (error of commission).

Abbreviations: HC, healthy control; IGD, internet gaming disorder; OCD, obsessive-compulsive disorder; IQ, intelligent quotient; Y-IAT-K, Korean version of the Young internet addiction test; BIS-11, Barratt impulsiveness scale version 11; BDI, Beck depression inventory; BAI, Beck anxiety inventory; Y-BOCS, Yale-Brown obsessive compulsive scale; NA, not applicable. Data are given as mean (standard deviation). **P < 0.005, *P < 0.05.

**Table 2:** Comparison of Go/Nogo-N2 amplitudes and latencies across three groups.

	HC	IGD	OCD	Statistical analysis^a^	
	(N = 26)	(N = 27)	(N = 24)	*F*	*Uncorrected P*
Go condition					
N2 amplitude (μV)/latency (ms) at F1	−2.9 (2.0)/243.9 (50.1)	−3.0 (2.2)/250.0 (45.5)	−2.6 (2.0)/270.6 (56.3)	0.165/1.904	0.848/0.156
N2 amplitude (μV)/latency (ms) at Fz	−3.3 (2.1)/250.5 (52.1)	−3.5 (2.0)/256.2 (42.5)	−2.9 (1.9)/269.8 (53.0)	0.595/1.009	0.554/0.370
N2 amplitude (μV)/latency (ms) at F2	−3.2 (2.1)/254.3 (53.4)	−3.1 (1.9)/258.6 (45.0)	−2.4 (1.8)/268.4 (49.0)	1.324/0.532	0.272/0.590
N2 amplitude (μV)/latency (ms) at C1	−1.2 (1.7)/188.4 (49.3)	−1.4 (2.0)/201.1 (55.3)	−1.4 (1.6)/216.1 (49.5)	0.054/1.801	0.948/0.172
N2 amplitude (μV)/latency (ms) at Cz	−1.0 (1.6)/193.4 (57.4)	−1.0 (2.4)/181.1 (53.7)	−1.1 (2.0)/203.0 (48.6)	0.043/1.132	0.958/0.328
N2 amplitude (μV)/latency (ms) at C2	−0.8 (1.5)/187.5 (56.4)	−0.8 (2.2)/185.1 (53.7)	−0.9 (1.8)/206.8 (50.1)	0.016/1.223	0.984/0.300
NoGo condition					
N2 amplitude (μV)/latency (ms) at F1	−3.6 (2.1)/241.2 (34.4)	−4.2 (2.2)/259.5 (26.6)	−2.9 (2.3)/256.3 (51.1)	2.054/1.697	0.135/0.190
N2 amplitude (μV)/latency (ms) at Fz	−4.3 (2.2)/252.0 (30.9)	−5.0 (2.5)/257.0 (19.2)	−3.1 (2.2)/261.0 (39.3)	4.438/0.542	0.015*/0.584
N2 amplitude (μV)/latency (ms) at F2	−4.0 (2.3)/256.5 (34.1)	−4.7 (2.1)/257.4 (22.1)	−2.7 (2.2)/258.3 (43.5)	5.065/0.017	0.006*/0.984
N2 amplitude (μV)/latency (ms) at C1	−2.1 (2.0)/205.2 (53.4)	−2.7 (1.7)/231.7 (45.6)	−3.8 (2.3)/216.3 (39.7)	0.947/2.143	0.393/0.125
N2 amplitude (μV)/latency (ms) at Cz	−3.0 (3.1)/196.7 (43.8)	−3.0 (2.3)/222.0 (32.4)	−2.7 (2.9)/218.5 (35.0)	0.120/3.477	0.888/0.036*
N2 amplitude (μV)/latency (ms) at C2	−2.7 (2.3)/202.2 (44.8)	−2.8 (1.9)/235.2 (30.5)	−2.2 (2.3)/221.3 (33.8)	0.521/5.347	0.596/0.007*

^a^Analysis of variance.

Abbreviations: HC, healthy control; IGD, internet gaming disorder; OCD, obsessive-compulsive disorder. Data are given as mean (standard deviation). *Uncorrected P < 0.05.

**Table 3:** Comparison of Go/Nogo-P3 amplitudes and latencies across three groups.

	HC	IGD	OCD	Statistical analysis^a^	
	(N = 26)	(N = 27)	(N = 24)	F	*Uncorrected P*
Go condition					
P3 amplitude (μV)/latency (ms) at C1	3.1 (2.1)/341.3 (58.6)	3.5 (2.9)/361.1 (57.5)	3.3 (2.6)/381.0 (36.5)	0.184/3.588	0.832/0.033*
P3 amplitude (μV)/latency (ms) at Cz	4.2 (2.4)/353.8 (63.3)	4.5 (2.7)/365.4 (55.7)	4.4 (3.8)/374.8 (43.4)	0.075/0.921	0.928/0.403
P3 amplitude (μV)/latency (ms) at C2	4.2 (2.0)/351.9 (55.9)	4.3 (2.7)/365.7 (51.4)	3.2 (3.3)/375.1 (53.2)	1.055/1.200	0.353/0.307
P3 amplitude (μV)/latency (ms) at P1	4.6 (2.2)/288.3 (26.6)	3.9 (1.8)/312.7 (43.5)	4.3 (1.6)/310.2 (27.3)	1.006/4.115	0.371/0.020*
P3 amplitude (μV)/latency (ms) at Pz	5.1 (2.2)/296.7 (30.1)	4.4 (1.9)/309.6 (40.2)	4.5 (2.0)/307.2 (28.8)	0.808/1.082	0.450/0.344
P3 amplitude (μV)/latency (ms) at P2	4.1 (2.4)/308.8 (50.2)	4.2 (1.6)/300.8 (35.5)	3.9 (2.1)/298.3 (27.8)	0.112/0.501	0.894/0.608
NoGo condition					
P3 amplitude (μV)/latency (ms) at C1	5.0 (3.2)/341.8 (45.0)	5.3 (4.0)/357.9 (42.2)	5.8 (3.9)/353.5 (30.9)	0.343/1.132	0.711/0.328
P3 amplitude (μV)/latency (ms) at Cz	6.7 (3.9)/338.2 (40.8)	6.2 (3.2)/365.5 (38.8)	6.8 (4.1)/347.5 (33.6)	0.220/3.540	0.803/0.034*
P3 amplitude (μV)/latency (ms) at C2	6.1 (3.1)/348.9 (37.0)	6.1 (3.6)/364.3 (38.1)	5.1 (3.8)/353.7 (30.5)	0.622/1.295	0.540/0.280
P3 amplitude (μV)/latency (ms) at P1	4.6 (2.0)/321.9 (51.3)	4.3 (1.6)/332.9 (65.5)	4.3 (2.0)/347.2 (59.7)	0.235/1.146	0.791/0.324
P3 amplitude (μV)/latency (ms) at Pz	4.9 (2.1)/315.8 (47.2)	4.7 (1.7)/351.5 (61.4)	4.3 (2.0)/338.3 (63.5)	0.540/2.581	0.585/0.083
P3 amplitude (μV)/latency (ms) at P2	4.5 (2.3)/320.3 (40.9)	4.5 (1.7)/329.8 (63.7)	4.0 (1.9)/323.8 (56.2)	0.637/0.207	0.532/0.814

^a^Analysis of variance.

Abbreviations: HC, healthy control; IGD, internet gaming disorder; OCD, obsessive-compulsive disorder. Data are given as mean (standard deviation). *Uncorrected P < 0.05.
